# Climatotherapy in Japan: a pilot study

**DOI:** 10.1007/s00484-017-1418-x

**Published:** 2017-08-04

**Authors:** Hitomi Kanayama, Yukinori Kusaka, Takayoshi Hirai, Hiroyuki Inoue, Yuko Agishi, Angela Schuh

**Affiliations:** 1 Environmental Health, Department of International Social and Health Sciences, Faculty of Medical Sciences, University of Fukui, 23-3 Matsuoka Shimoaizuki, Eiheiji-cho, Yoshida-gun, Fukui, 910-1193 Japan; 2 Faculty of Nursing and Social Welfare Sciences, Fukui Prefectural University, Fukui, Japan; 3 Faculty of Global and Community Studies, University of Fukui, Fukui, Japan; 4 Research Institute of Health Resort Medicine, Tokyo, Japan; 5 Public Health and Health Services Research, Department of Medical Informatics, Biometry and Epidemiology, Ludwig-Maximilians-University (LMU) Munich, Munich, Germany

**Keywords:** Climatotherapy, Mountain, Blood pressure, Heart rate, Anger, Profile of Mood Status

## Abstract

Twenty-nine urban inhabitants participated in a half-day climatotherapy programme at the moderate mountain area and lowland area in the northwest part of the main island of Japan. The current study was aimed to investigate physically and mentally the objective and subjective influence of our short programme, which was a prospective pilot study of single intervention. Blood pressure was significantly descended during terrain cure at the uphill mountain path and returned after fresh-air rest cure, while there was no significant change throughout the programme at lowland flat path. Heart rate was significantly ascended and descended at both area, and more clearly changed at the mountain path. Profile of Mood Status brief form Japanese version administered before and after our half-day programme. Age adjusted *T* score of negative subscales, ‘tension-anxiety’, ‘depression’, ‘anger-hostility’, ‘fatigue’ and ‘confusion’ were significantly lower after climatotherapy at both sites. Whereas, there was no significant change concerning ‘vigour’ score. This short-version climatotherapy programme has been designed for people without enough time for long stay at health resort. It turned out our half-day climatotherapy programme contribute to mood status improvement. In addition, repeated practice of our short-version programme including endurance exercise with cool body shell using uphill path can be expected that blood pressure will go toward the normal range and heart rate will decrease both in usual time and during exercise. Therefore, health benefits can be expected of this climatotherapy programme.

## Introduction

Climatotherapy is conducted in a marine climate or in upland and mountain regions (Schuh 1993). The climatic terrain cure, endurance walking exercise on uphill or sandy paths (terrain cure) under slightly cool condition, is the main part of the climatotherapy, and especially cold air and UV radiation fulfil an important role.

In Europe, scientific evidence of long-lasting effects of several-weeks’ climatotherapy has been provided for atopic diseases, lack of exercise, hypertension, metabolic syndrome and heart and circulatory diseases in high altitude, for atopic diseases in moderate altitude, and for atopic diseases, lack of exercise and heart and circulatory diseases at the North Sea (Schuh and Nowak 2011).

Because Japan consists of over 60% of forests and mountain areas (Statistics Bureau, Ministry of Internal Affairs and Communication 2016), Shinrin-yoku (forest-air bathing/walking) is popular. There are many studies about the effectiveness (cerebrovascular functions, immunological markers, mental health status) of Shinrin-yoku (Ohtsuka et al. 1998; Morita et al. 2007; Tsunetsugu et al. 2010). Climatotherapy in moderate altitude mostly includes Shinrin-yoku, because of tree-rich environment. On the other hand, climatic factors are not in the focus of Shinrin-yoku.

Japanese people today seldom take sufficient time (Sasaki et al. 2007). Germans have also become pressed for time in recent years. Based on the German climatotherapy method, we designed a short climatotherapy programme for busy Japanese people, which combines climatic terrain cure with fresh-air rest cure (resting outside under slightly cool conditions) within a half-day.

This study was a prospective pilot study of a single intervention and aimed to examine the influence of this programme on participants’ physical and mood status.

## Materials and methods

Urban inhabitants were recruited as participants in the spring of 2014, 2015 and 2016. Our study sites, Yatsusugi Forest in the moderate mountain and, as a reference site, Fukui Prefectural General Green Center (Green Center) in the lowland, were located in the northwest part of the main island of Japan. Yatsusugi Forest path was consisted of several uphill and downhill. Green Center path in a tree-rich park was almost flat. All subjects participated our short-version climatotherapy during the morning at both sites on different 2 days.

Each participant was instructed to maintain his/her own subjective temperature under ‘slightly cool conditions’ to meet the climatotherapy purpose.

Height, body weight and body fat percentage data were collected. Systolic blood pressure (SBP), diastolic blood pressure (DBP) and heart rate (HR) were measured using portable automatic blood pressure and heart rate monitors.

Physical Condition and Exercise Habit Questionnaire was adopted from the ‘National Health and Nutrition Survey (NHNS)’ questionnaire (Ministry of Health, Labour and Welfare 2014).

POMS (Profile of Mood Status) brief form Japanese Edition is composed of six subscales: Tension-Anxiety (T-A), Depression-Dejection (D), Anger-Hostility (A-H), Vigour (V), Fatigue (F) and Confusion (C). A standardized score by gender and age group (*T* score) was used because of age disparities among the participants.

Wilcoxon signed-rank test was used for paired groups, and the Friedman test was used for three or more groups. Bonferroni correction was performed for multiple comparisons. A two-tailed *p* value of less than 0.05 was considered significant.

Our study received ethical approval from the ethics committee of the institutional review board (IRB) of the University of Fukui.

## Results

The mean age of 29 participants (17 males, 12 females) was 66.0 (65.8 in males, 66.2 in females). The mean values of height, weight, BMI and body fat percentage of participants were similar to the NHNS (2014) data. There were no other great differences in the percentage of medications between participants and the NHNS (2014) data except the anti-hypertensive drug medication was remarkably lower in females. The percentages of regular exercise habits were remarkably higher among 24 elderly participants (≥ 60 years) compared to the corresponding NHNS (2014) data. By contrast, only three participants under 49 years reported no regular exercise habits.

SBP significantly declined from 141 to 119 mmHg (*p* < 0.001) during the climatic terrain cure at the mountain site with a coincidence of the significant HR increase from 72 to 101 bpm (*p* < 0.001). BP at the flat site was not significantly altered. More than three times the participants reached the target exercise strength of 50% (targetHR_50%_) at the mountain site than at the flat lowland site.

On both sites, *T* scores of five negative mood profiles improved significantly (T-A −8, *p* < 0.001; D −3, *p* = 0.001; A-H −8, *p* < 0.001; F −4, *p* = 0.007; C −3, *p* = 0.003 at the mountain site).

## Discussion

Changes of SBP, DBP and HR were acute physiological reactions to exercise in general; no endurance increase can be expected. Previous studies showed at rest and during exercise, SBP and HR decreased 5–10% after a 3- or 4-week climatic terrain cure (Inama and Halhuber 1975; Schuh 1997).

We instructed the participants to self-regulate their subjective temperature of feeling ‘slightly cool’. The participants could only experience the cool body shell effect once. Schuh (1993) demonstrated that a terrain cure with slightly cool skin temperature will lead to an increase of aerobic endurance as well as hardening. Therefore, the implementation of a cool regime of the skin during training leads to a health promoting effect, which can be easily adapted in daily life.

But different factors led to an insufficient cool regime: (1) participants did not completely understand the climatotherapy concept; (2) the on-site temperature was too high to perform exercises; and (3) participants were not allowed to use alternative cooling methods.

Ten participants reached the targetHR_50%_, which demonstrated the advantage of the mountain site (Yatsusugi Forest). Most participants were elderly, and their lifestyle was already active. Nevertheless, one single climatotherapy intervention can hardly create an increase in endurance capacity. But regular endurance training with a cool body shell will nearly double the training effects caused by an additional cold-induced increase of the aerobic muscle metabolism (Schuh 1993).

Li et al. (2007) reported that 2 of 5 negative mood statuses and one positive mood status (vigour) were significantly improved after forest bathing. Alike this, all negative mood statuses of the participants were significantly improved—except vigour—after climatotherapy. Tree-rich environment is common at Shinrin-yoku sites as well as on both study sites. Most participants were deeply relaxed and fell asleep during the fresh-air rest cure after the climatic terrain cure. This may be the reason why vigour kept unchanged at both sites.

Our study shows several limitations. First, this was a non-randomized pilot study. In general, active and vigorous persons are likely to register faster. Therefore, if the general elders participated, then our programme could produce a greater change. Additionally, the sample size of 29 participants was not sufficiently enough and a control group was missing.

We may expect on the long-term the normalization of BP and HR, sustaining of good mood status in participants who acquire healthy behaviours and continue self-practice based on the current programme. Our programme appears to be of potential value for the physical and mental health of busy people to resist daily work stressors when exercising in mountain areas. Additionally, a cool regime of the skin is easy to adapt to relieve daily stress. Further studies should be done to observe long-term health benefits of a short-term climatotherapy programme.
